# Spatial complexity enhances predictability in food webs

**DOI:** 10.1038/srep43440

**Published:** 2017-02-27

**Authors:** Akihiko Mougi

**Affiliations:** 1 Department of Biological Science, Faculty of Life and Environmental Science, Shimane University, 1060 Nishikawatsu-cho, Matsue 690-8504, Japan

## Abstract

The prediction of an ecosystem’s response to an environmental disturbance or the artificial control of ecosystems is a challenging task in ecology. Ecological theory predicts that disturbances frequently result in unexpected responses between interacting species due to the many indirect interactions within a complex community. However, such indeterminacy appears to be unusual in nature. Here using a meta-community food web, I show that spatiality is key to resolving this disparity. A moderate level of spatial coupling strength between habitats due to species migration increases the possibility of expected responses to press perturbation or predictability. Moreover, predictability increases with increasing spatial complexity, as measured by the number of local food webs and their connectivity. A meta-community network can attenuate the propagation of disturbances through indirect pathways due to species emigration to other habitats, thereby preserving the expected effect on the interacting species. These results suggest that the isolation of communities due to habitat destruction decreases the predictability of communities, thereby complicating the control of ecosystems.

How to predict species responses to environmental disturbances is a long-standing goal in ecology^1^,^2^. Earlier theories have suggested that even a qualitative prediction of species responses to a perturbation is challenging^3^,^4^,^5^,^6^,^7^,^8^. An increase in the abundance of predators does not necessarily decrease the abundance of prey, contrary to the expected direct effect of a predator on prey. Such low predictability results from many indirect pathways through which perturbations are dampened or magnified, masking or even counteracting the direct effect^3^. This complexity within ecosystems and low resolution of quantifying interaction strengths limit our ability to understand community dynamics because inherent estimation errors in interaction strengths can easily reverse the direction of species responses to a perturbation. However, contrary to theoretical predictions, natural ecosystems appear to frequently show expected responses^9^,^10^,^11^,^12^ (but see refs 13 and 14). An increase in sea otters decreases sea urchins, whereas a reduction in wolves increases elk. These expected patterns are widely consistent across various regions and different communities. This contradiction between theory and natural observations suggests that ecosystems possess a mechanism to avoid distractions from indirect effects^10^,^15^,^16^,^17^.

Weak interactions^18^ result in an improved predictability^16^,^17^,^19^. Recent studies have considered realistic allometrically constrained parameterization and a non-linear functional response in a food web model to show that increasing network complexity can increase the predictabilities of interaction strengths or species responses to sustained press^17^ or pulse^16^,^19^ perturbations. These common results are expected to originate from a mechanism where interaction strengths become weaker with an increasing network size due to interaction effort allocations, where weaker interactions reduce propagations in indirect pathways^10^,^15^. Such earlier studies have considered non-spatial structured food webs. Here I propose a novel mechanism for enhancing predictability in food webs, i.e., spatiality, as a general and inherent feature of any natural ecosystem.

Ecological communities are composed of sub-local communities connected by species migration^20^. In this context, earlier studies were limited to isolated local communities. However, even if we are interested in the effects of perturbation on such a specific local community, the meta-community structure may affect the predictability in the local community because perturbation can propagate to other local communities through species migration. In the present study, I aim to demonstrate that spatiality contributes to our understanding of how disturbance affects food web predictability.

Consider a “meta-food web” in which organisms randomly move between numerous local food webs^21^. Food webs and interaction networks are comprised of *N* species, and their interaction links are determined by the probability of a pair of species being connected by a trophic link, *C*. Meta-food webs and habitat networks are comprised of *H*_*N*_ patches and the proportion of food-web pairs between which an organism can move, *H*_*C*_. I define spatial complexity by *H*_*N*_ and *H*_*C*_. The strength of coupling of local food webs is given as *M* (see Methods). Random networks are assumed for both food web and habitat structure. I conduct press perturbations^3^,^4^ and the long-term effects of small disturbances on species abundances (e.g., effects of fisheries harvest, pollution, eutrophication, or a controlled experiment) and reveal the net effects (effects of all direct and indirect interactions) of such disturbances on equilibrium species abundances. Predictability is evaluated based on the proportion of true predator (or prey) net responses matching the sign of net effects after introducing an error in interaction strength estimates. For each given network and error level, I calculated the mean proportion of net responses matching the signs of true net responses over many random iterations (Methods). I control *M, H*_*N*_, and *H*_*C*_ to examine the effects of spatiality on predictability.

## Results/Discussion

**Figure 1:**
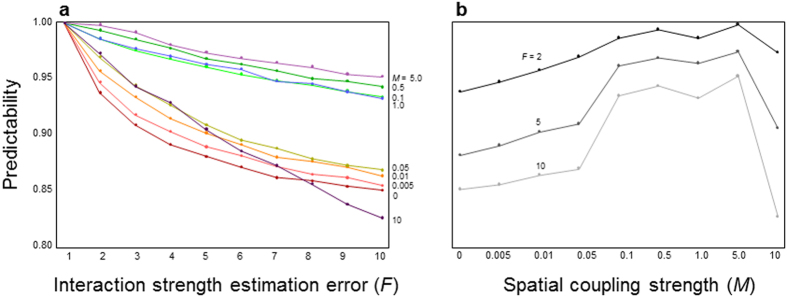
Effects of estimation error (*F*) on predictability with varying spatial coupling strength. (**a**) Negative relationships between predictability and estimation error. (**b**) Unimodal relationships between predictability and spatial coupling strength*. N* = 20, *C* *=* 0.15, *H*_*N*_ = 12, and *H*_*C*_ = 0.6.

**Figure 2:**
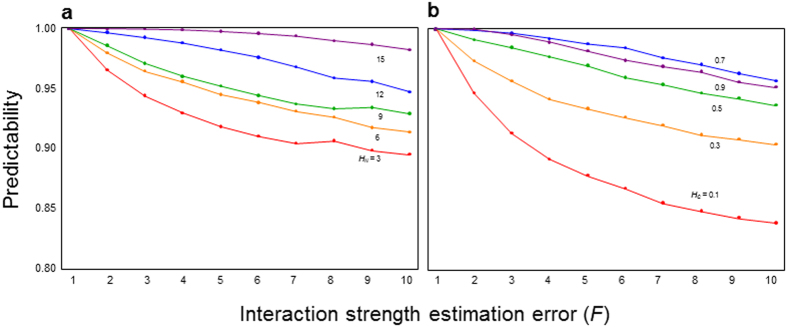
Relationships between estimation error (*F*) and predictability with varying spatial complexity. (**a**) Effect of habitat number (*H*_*N*_), where *H*_*C*_ = 0.6. (**b**) Effect of habitat connectivity (*H*_*C*_), where *H*_*N*_ = 12. Colors indicate different levels of habitat number and connectivity. *N* = 20, *C* = 0.15, and *M* = 1.

Consider a case where the local food webs are isolated (*M* = 0). As shown by earlier theoretical studies^3^,^4^,^5^,^6^,^7^,^8^, predictability in focal local food webs is not high, particularly when the estimation error is large (Fig. 1a). However, when local food webs are coupled with migration (*M* > 0), predictability in focal local food webs increases and reaches its peak predictability at a moderate coupling strength (Fig. 1b). Furthermore, predictability depends on spatial complexity, *H*_*N*_, and *H*_*C*_. When local food webs are loosely coupled (intermediate *M*), predictability tends to increase with an increasing number of local food webs, *H*_*N*_ (Fig. 2a), or increasing connection probability, *H*_*C*_ (Fig. 2b). However, when the coupling between local food webs is too weak (smaller *M*) or too tight (larger *M*), predictability shows no clear relationship with spatial complexity (Fig. S1). This response suggests that high predictability arising from local food-web coupling is stronger in spatially complex ecosystems and that predictability will not arise when the coupling is so strong that the entire meta-food web behaves as a single food web. Furthermore, given weakly connected interaction links observed in empirical food webs, predictability markedly increases (Fig. S2). These results remain qualitatively unchanged by changing the network type of a food web (random or cascade) (Fig. S3) or perturbed species (predator or prey) (Fig. S4).

**Figure 3:**
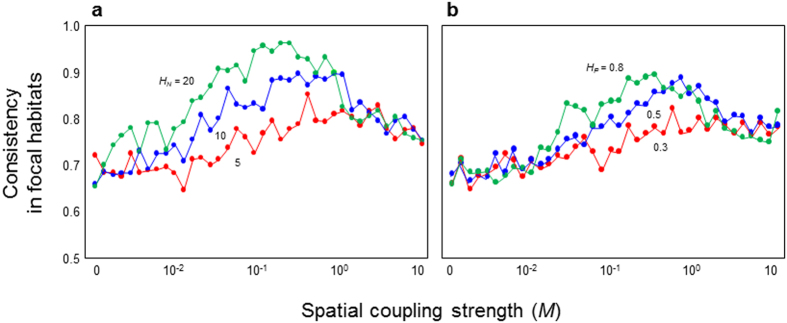
Relationships between spatial coupling strength (*M*) and consistency. (**a**) Effect of habitat number (*H*_*N*_), where *H*_*C*_ = 0.6. (**b**) Effect of habitat connectivity (*H*_*C*_), where *H*_*N*_ = 8. Colors indicate different levels of habitat number and connectivity. *N* = 20 and *C* = 0.5.

The improvement of predictability due to spatiality suggests that spatial network and species migration have a buffering effect on perturbation through interaction pathways. In fact, the recovery rate or resilience to perturbation tends to be high when the predictability is high (Fig. S5). It is still unclear what causes the high resilience in this system. I hypothesized that spatiality weakens the magnitudes of indirect effects, allowing food webs to be highly resilient. To reveal whether spatiality weakens indirect effects, I examined a relationship between spatiality and the degree of coincidence in directions between direct and net species responses to perturbation (consistency) (see Methods). If indirect effects are weakened by spatiality, net species responses to perturbation should show more consistent directions with those expected from direct responses. In fact, Fig. 3 shows this expected pattern. The consistency is likely to be high under a moderate coupling strength. In such conditions, spatial complexity (*H*_*N*_ and *H*_*C*_) tends to increase consistency (Fig. 3). In addition, weaker interaction links observed in empirical food webs increase consistency (Fig. S6).

**Figure 4:**
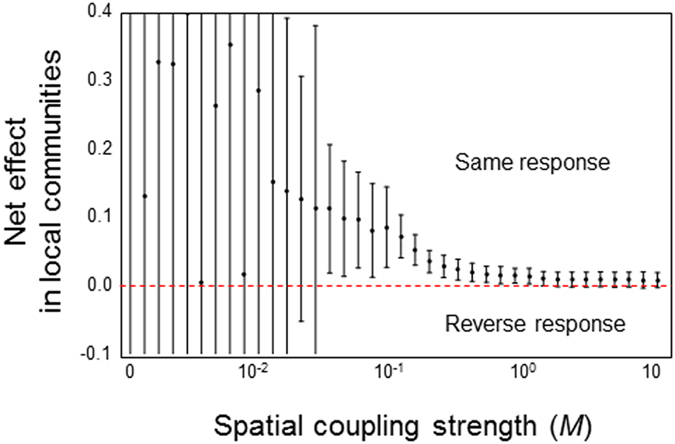
Relationships between spatial coupling strength (*M*) and net effects in local communities. Black points indicate mean values. Black lines are error bars. In this plot range, the full error bars, including the lower values of *M*, are not shown. The red dashed line indicates the zero line. Values greater than zero indicate a positive response to perturbation (the directions of net and direct response are the same). Parameters are the same as those in Fig. 3a.

The increase in consistency due to spatiality can be explained by the buffering effect of perturbation through species emigration to other habitats. In isolated food webs, net species responses to perturbation inherently show a reversal direction expected from direct responses because many indirect effects can mask or counteract the direct effect (lower *M* in Fig. 4). However, if a focal local food web is connected to other local food webs, perturbation can propagate not only to indirect pathways within the focal local food web, but also to other habitats through species emigration. By allowing individuals to move, the effects of perturbation can attenuate through indirect pathways. Consequently, the species net responses reflect the direct effects (moderate *M* in Fig. 4). However, if local food webs are tightly coupled, perturbation strongly propagates to other habitats and easily returns to the focal local food web. This results in a narrowing of the difference in consistencies between focal and non-focal local food webs (Fig. 3a and Fig. S7), thereby intensifying the indirect effects and masking or counteracting the direct effects (higher *M* in Fig. 4).

Yodzis theoretically showed that predicting the responses of species to press perturbation is inherently difficult due to the indirect pathways within a community^3^,^4^. Recent theories have provided some ideas for improving predictability. Availability to precise information regarding parameters related to interaction strengths allows accurate prediction^5^,^7^,^8^. However, in real complex ecosystems, such predictions are practically impossible because errors in the estimations of many parameters quickly reduce the accuracy of predictions^7^.

The present study showed that complexity of a habitat network largely improves qualitative predictability of species responses to press perturbation. This suggests that natural complex ecosystems comprised of diverse habitats are not likely to show unexpected responses to environmental disturbances. If this is indeed the case, the artificial control of ecosystems may be less difficult than expected. Furthermore, the result showed that small changes in the interaction strength do not qualitatively alter population responses to perturbation, suggesting that population responses are consistent over time even if interaction strengths are fluctuating. Spatiality may make ecosystem responses robust to the fluctuation of both species abundances and interaction strengths. However, less optimistic viewpoints exist: (1) more complex communities with many species and/or interaction links require more complex spatiality with many habitats and/or habitat links to maintain higher predictability, (2) the press perturbation theory assumes locally stable equilibrium species abundances, and (3) in the present study, perturbation occurred only locally and not regionally, thereby leaving the question of how global environmental changes such as global warming affect the predictability of spatial ecosystems unanswered. To counter the first pessimistic viewpoint, real ecosystems may maintain higher predictability because complex communities with diverse species are expected to be supported in diverse habitats. In fact, a recent theory predicts that complex communities are not maintained in few habitats and require a more complex habitat network with many habitats and habitat links^21^.

Habitat destruction and modifications can possibly not only reduce the stability of ecosystems^21^,^22^,^23^,^24^, but also result in species responses to environmental disturbances being difficult to predict. Habitat destruction can decrease the number of local food webs (lower *H*_*N*_), and disconnect pairs of local food webs by restricting movement of animals between habitats (lower *H*_*C*_). Any of these changes have the potential to reduce predictability in ecosystems. Considered collectively, we may need to maintain ecosystems to predict how a decline in any species or species losses due to environmental destruction changes ecosystems.

## Methods

Consider a meta-food web model^21^. The model assumes a random (or cascade in Fig. S3) food web in which each pair of species, *i* and *j (i, j* = 1, …, *N*), is connected by a trophic interaction with probability *C*. The maximum link number, *L*_max_, is *N(N* − 1)/2. The spatial food web model is defined using the following ordinary differential equation:


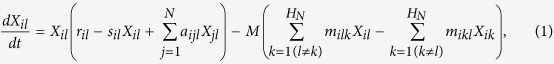


where *X*_*il*_ (*l* = 1…*H*_*N*_) is the abundance of species *i* in habitat *l, r*_*il*_ is the intrinsic rate of change of species *i* in habitat *l, s*_*il*_ is the density-dependent self-regulation of species *i* in habitat *l*, and *a*_*ijl*_ is the interaction coefficient between species *i* and species *j* in habitat *l*. Interaction coefficients are defined as *a*_*ijl*_ = *e*_*ijl*_*α*_*ijl*_ and *a*_*jil*_ = −*α*_*ijl*_, where *α*_*ijl*_ is the consumption rate and *e*_*ijl*_ (<1) is the conversion efficiency. The migration rate is the product of a scaling parameter, *M* (spatial coupling strength), and the species-habitat specific migration rate, *m*_*ilk*_, where *k* = 1 … *H*_*N*_ but *k* ≠ *l*. For simplicity, *m*_*ilk*_ = *m*_*ikl*_ is assumed. *s*_*il*_ is set to 1. Equilibrium species abundance 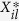 and parameters *e*_*ijl*_, *α*_*ijl*_, and *m*_*ilk*_ are randomly chosen from a uniform distribution, U[0, 1]. *r*_*il*_ is calculated such that *dX*_*il*_/*dt* = 0 for all *i* and *l*^21^,^25^.

Consider the Jacobian matrix (J) that represents the pair-wise partial derivatives of the growth rate of the *i*th species in the *l*th habitat with respect to the population size of species *j* in the *k*th habitat. Submatrices of the Jacobian matrix (J), *J*_*ij*_, are *N* × *N* matrices. Diagonal submatrices, *J*_*jj*_, are Jacobian matrices of *j*-th local food web in isolation. Each element in the diagonal submatrices of the Jacobian matrix *J*_*ii*_ represents the direct effect between two species within a same habitat (*k* *=* *l*) by which a small increase in species *j* affects the population growth rate of species *i.* The diagonal and off-diagonal elements are represented as 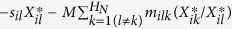 and 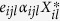 (when sp. *i* consumes sp. *j*) or 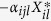 (when sp. *j* consumes sp. *i*), respectively. The off-diagonal submatrices *J*_*ij*_ (*i* ≠ *j*) are the Jacobian matrices representing the effect of movements between local food webs. The diagonal and off-diagonal elements are *Mm*_*ilk*_ and zero, respectively. Net effects between two species within a same habitat, and combined effects of direct and indirect effects, are represented by *ij*th elements in the submatrices of the negative inverse Jacobian matrix (−J^−1^)^3^. The existence of the inverse matrix is guaranteed by the stability of the matrix J. Each element in the diagonal submatrices of −J^−1^, 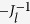 reflects the direction and relative magnitude by which the local equilibrium abundances of a species in row *i (X*_*il*_) will respond to a sustained small input of individuals to the species in column *j (I*_*j*_) because


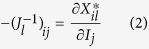


is obtained by differentiating equations, *f*_*i*_(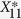, ….) = 0 (for all *i* ≠ *j*) and *f*_*j*_(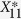, ….) + *I*_*j*_ = 0 with respect to *I*_*j*_^3^,^4^. As 
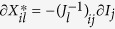
, a small press perturbation, *I*_*j*_, will cause a change in the equilibrium abundance of species *i* that is proportional to 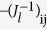.

Having a Jacobian matrix constructed by the above procedures, I calculated predictability based on the proportion of true net responses in −J^−1^ matching the sign structure of net effects after varying levels of error were introduced to the elements of J^3^,^7^. Error in interaction strength estimates was added to each interaction value of J. For each *a*_*ijl*_, I randomly drew new values either from an underestimate, uniform distribution (*a*_*ijl*_/*F, a*_*ijl*_), or an overestimate, uniform distribution (*a*_*ijl*_, *Fa*_*ijl*_), of each element of J, where *F* represents the maximum possible proportional error of an error, which varied from 1 (no error) to 10 (an order-of-magnitude error)^7^. For each given network and error level, I calculated the mean proportion of predator (or prey in Fig. S4) net responses matching the signs of the true net responses over 500 random iterations.

I calculated consistency as the probability of the net responses of a predator to randomly selected perturbed prey in −J^−1^ that matched the sign of the direct responses of the predator to the focal perturbed prey in J over 500 sample communities (in each calculation, mean direct and net effect of local communities are sampled). In a similar way, we can examine the consistency in different habitats (*k* ≠ *l*) by calculating net effects of a species *j* in the *k*th habitat to species *i* in the *l*th habitat (Fig. S7).

## Additional Information

**How to cite this article:** Mougi, A. Spatial complexity enhances predictability in food webs. *Sci. Rep.* **7**, 43440; doi: 10.1038/srep43440 (2017).

**Publisher's note:** Springer Nature remains neutral with regard to jurisdictional claims in published maps and institutional affiliations.

## Supplementary Material

Supplemental Figures
