# Supplementary material for: Spatial complexity enhances predictability in food webs
Source: Sci Rep. 2017 Feb 27;7:43440. doi: 10.1038/srep43440 (PMC5327493; doi:10.1038/srep43440)

Supplementary Information

Spatial complexity enhances predictability in food webs

A. Mougi

Supplemental Figures

Fig. S1. Relationships between estimation error (F) and predictability with varying spatial complexity. (a, c) Effect of habitat number (H_N), where $H_P = 0.6$. (b, d) Effect of habitat connectivity (H_C), where $H_N = 12$. I assumed $M = 10^{-3}$ in (a, b) and $M = 10$ in (c, d). Colors indicate different levels of habitat number and habitat connectivity. $N = 20$ and $C = 0.15$.

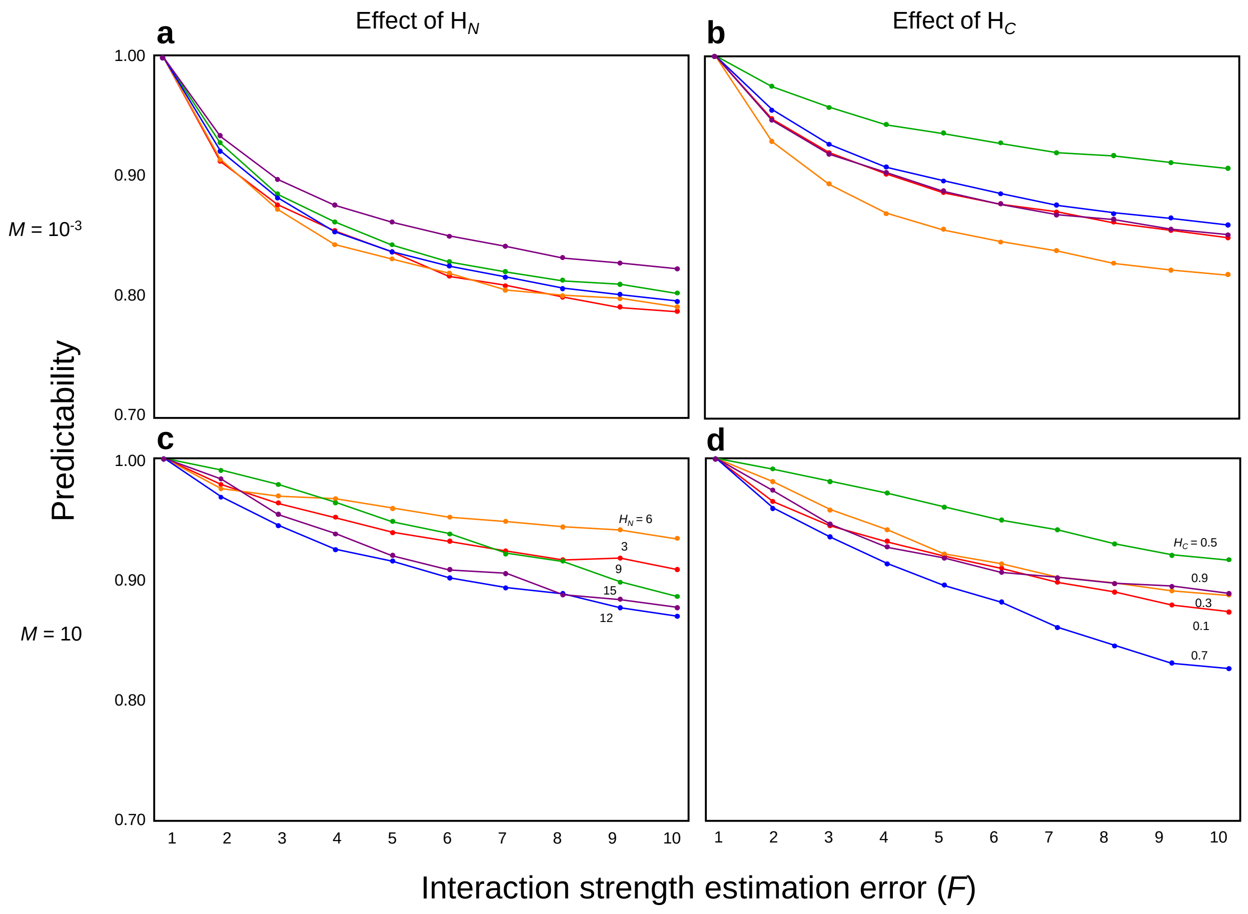

Fig. S2. Relationships between estimation error (*F*) and predictability with varying proportions of connected pairs (*C*). N = 20, M =1, H_N = 12 and H_C = 0.6.

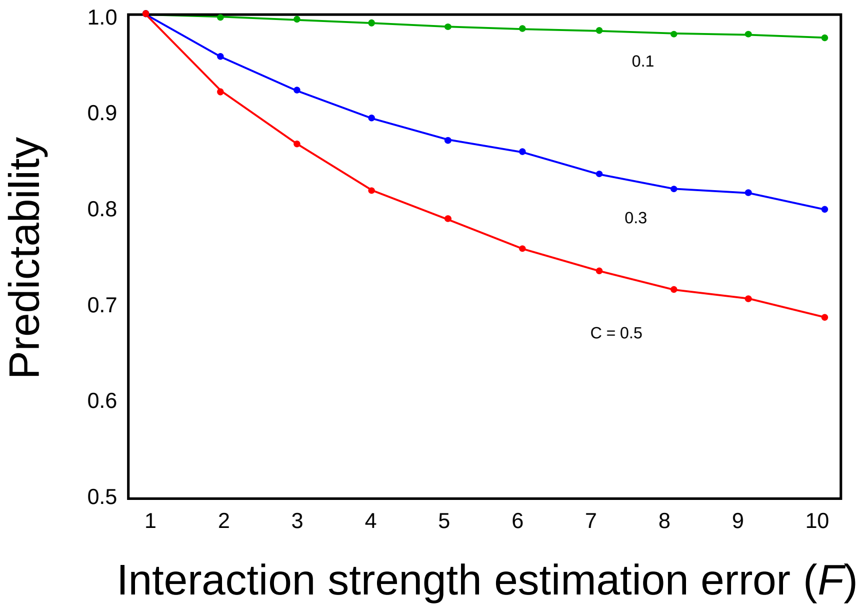

Fig. S3. Effects of spatiality to predictability in a cascade model. In the cascade model, for each pair of species, i, j = 1,…, n with $i < j$, species i never consumes species j, whereas species j may consume species i. (a) Relationships between spatial coupling strength and predictability with varying estimation error. (b) Effect of habitat number (H_N), where H_C = 0.6. (c) Effect of habitat connectivity (H_C), where H_N = 12. N = 20, C = 0.15 and M = 0.5.

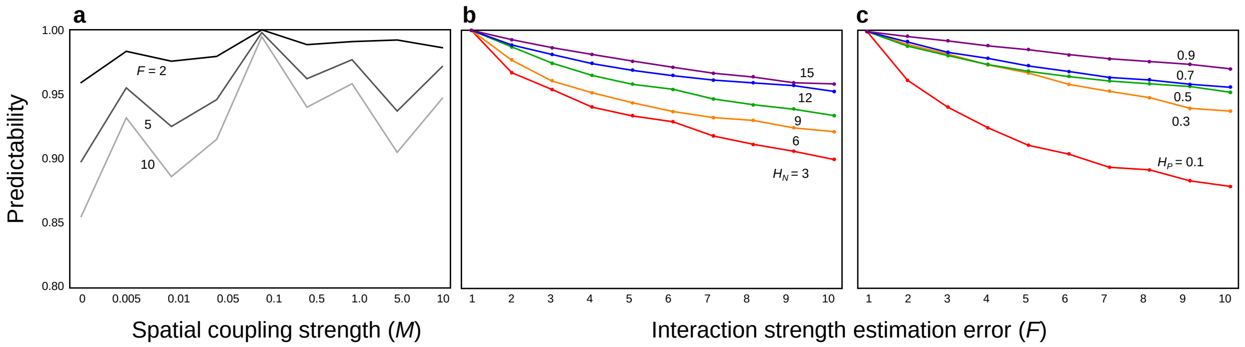

Fig. S4. Effects of spatiality to predictability in prey responses to a perturbation. The predictability is defined as the mean proportion of prey net responses that matched the signs of the true net responses over 500 random iterations. (a) Relationships between spatial coupling strength and predictability with varying estimation error. (b) Effect of habitat number (H_N), where H_C = 0.6. (c) Effect of habitat connectivity (H_C), where H_N = 12. N = 20, C = 0.15 and M = 0.5.

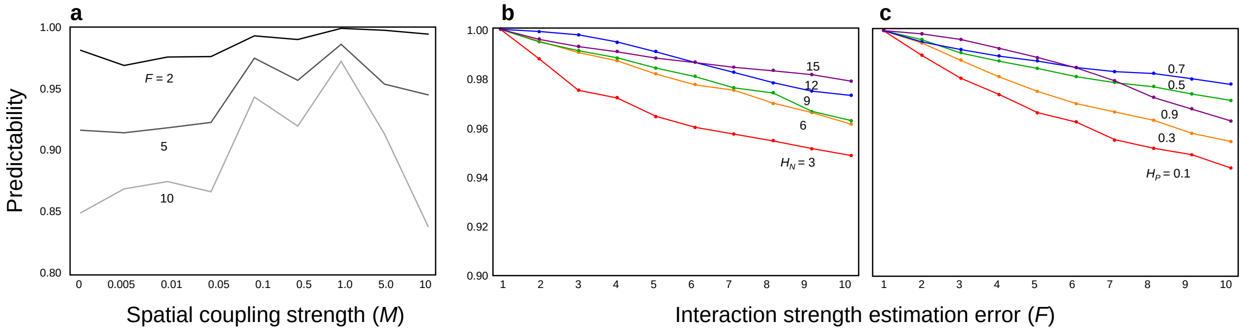

Fig. S5. Relationships between spatial coupling strength (*M*) and resilience. (a) Effect of habitat number (H_N), where H_C = 0.6. (b) Effect of habitat connectivity (H_C), where H_N = 8. Resilience, the capacity of the system to return to an equilibrium after a short and small disturbance, was calculated as the absolute value of the greatest real part of eigenvalues of the Jacobian matrix. Colors indicate different levels of habitat number and habitat connectivity (see Fig. 3). Other information is the same as that in Fig 3 in the main text.

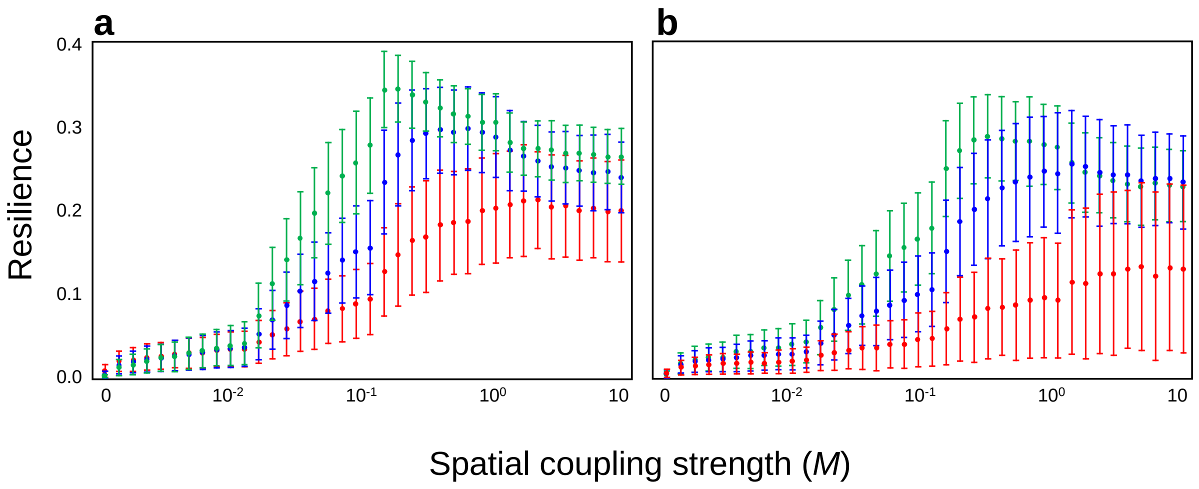

Fig. S6. Relationships between spatial coupling strength (*M*) and consistency with varying proportions of connected pairs (*C*). N = 20, H_N = 20, and H_C = 0.6.

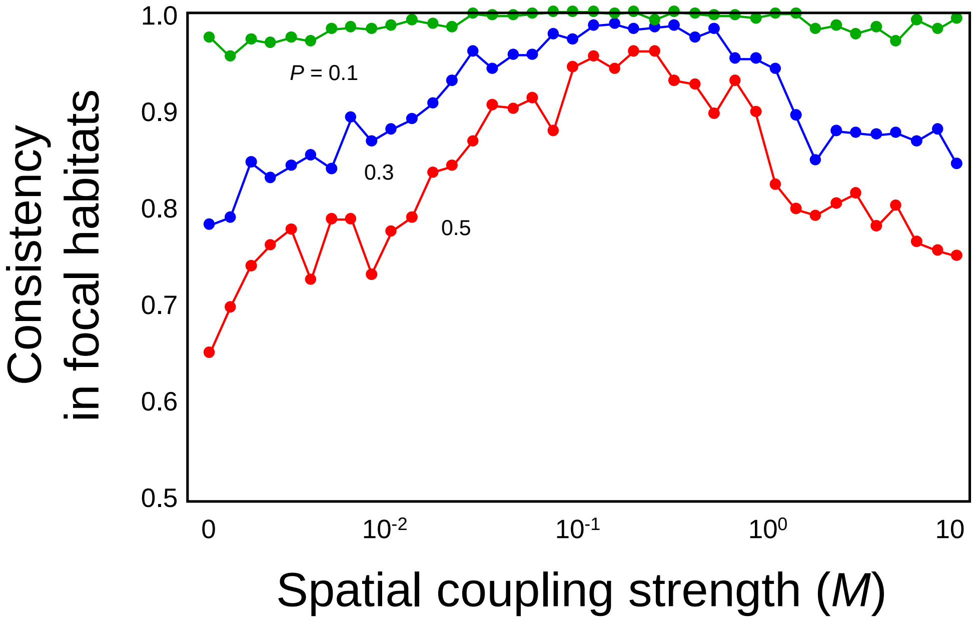

Fig. S7. Relationships between spatial coupling strength (*M*) and consistency in non-focal habitats with varying habitat number (H_N). Consistency is calculated by the probability of the net responses of the predator in non-perturbed habitats to the perturbed prey in a focal habitat $–J^{-1}$ that matched the sign of the direct responses of the predator to the focal perturbed prey in J over 500 sample communities. Colors indicate different levels of habitat number. Other information is the same as that in Fig. 3a in the main text.

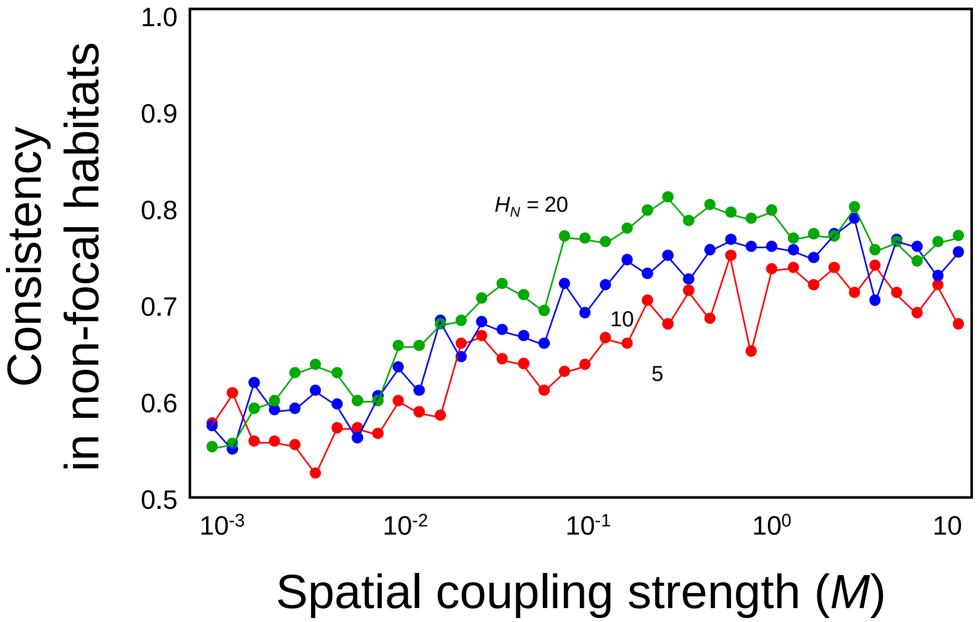

Supplement: Supplemental Figures [file srep43440-s1.pdf]
